# 5,7-Dimethoxyflavone Attenuates Sarcopenic Obesity by Enhancing PGC-1α–Mediated Mitochondrial Function in High-Fat-Diet-Induced Obese Mice

**DOI:** 10.3390/nu17162642

**Published:** 2025-08-14

**Authors:** Changhee Kim, Mi-Bo Kim, Sanggil Lee, Jae-Kwan Hwang

**Affiliations:** 1 Department of Biotechnology, Yonsei University, Seoul 03722, Republic of Korea; changgml@gmail.com; 2 Graduate Program in Bioindustrial Engineering, College of Life Science and Biotechnology, Yonsei University, Seoul 03722, Republic of Korea; mibokim1120@gmail.com; 3 Department of Food Science and Nutrition, College of Fisheries Science, Pukyong National University, Busan 48513, Republic of Korea; 4 Department of Smart Green Technology Engineering, Pukyong National University, Busan 48513, Republic of Korea

**Keywords:** 5,7-dimethoxyflavone, sarcopenic obesity, PGC-1α, muscle atrophy

## Abstract

**Background/Objectives**: Sarcopenic obesity, defined by the coexistence of excessive fat accumulation and progressive muscle loss, is associated with an increased risk of metabolic dysfunction and physical disability. While 5,7-dimethoxyflavone (DMF), a bioactive flavone derived from *Kaempferia parviflora*, has demonstrated anti-obesity and muscle-preserving properties, its effects on sarcopenic obesity remain unclear. **Methods**: Four-week-old male C57BL/6J mice were fed a high-fat diet (HFD) for 6 weeks to induce sarcopenic obesity, followed by 8 weeks of continued HFD with the oral administration of DMF. Muscle function was assessed through grip strength and treadmill running tests, while muscle and fat volumes were measured using micro-CT. Mechanistic analyses were performed using gene expression and Western blot analysis. **Results**: DMF significantly reduced body weight, fat mass, and adipocyte size while enhancing grip strength, endurance, skeletal muscle mass, and the muscle fiber cross-sectional area. In the gastrocnemius muscle, DMF increased the gene expression of *peroxisome proliferator-activated receptor gamma coactivator-1α* (*Ppargc1a*) and its isoform *Ppargc1a4*, thereby promoting mitochondrial biogenesis. It also improved protein turnover by modulating protein synthesis and degradation via the phosphatidylinositol 3-kinase/protein kinase B/mechanistic target of rapamycin signaling pathway. In subcutaneous and brown adipose tissues, DMF increased mitochondrial DNA content and the expression of thermogenic and beige adipocyte-related genes. These findings suggest that DMF alleviates sarcopenic obesity by improving mitochondrial function and regulating energy metabolism in both skeletal muscle and adipose tissues via PGC-1α-mediated pathways. Thus, DMF represents a promising therapeutic candidate for the integrated management of sarcopenic obesity.

## 1. Introduction

Obesity is a chronic metabolic disorder caused by an imbalance between energy intake and expenditure [1,2]. It is associated with a range of metabolic abnormalities, including insulin resistance, dyslipidemia, and type 2 diabetes [3]. Especially, sarcopenic obesity, defined by the concurrent presence of excess fat and progressive skeletal muscle loss, has emerged as a more complex condition that not only exacerbates metabolic disturbances but also increases the risk of physical disability, frailty, and mortality [4,5]. Unlike age-related sarcopenia, sarcopenic obesity is primarily driven by adipose-tissue-induced inflammation, oxidative stress, and impaired muscle metabolism, underscoring the pathological crosstalk between fat and muscle tissues [6].

Skeletal muscle plays a central role in systemic energy metabolism, accounting for approximately 40% of total body mass and up to 75% of total body protein [7]. It is also a major site for glucose disposal and fatty acid oxidation, thereby serving as a key regulator of whole-body metabolic homeostasis [8]. In obesity, however, muscle mass and quality are frequently impaired due to chronic low-grade inflammation, mitochondrial dysfunction, and disrupted protein turnover [9]. Peroxisome proliferator-activated receptor gamma coactivator-1 alpha (PGC-1α) is a key transcriptional coactivator that orchestrates mitochondrial biogenesis, oxidative metabolism, and muscle fiber type specification [10]. Among its isoforms, PGC-1α1 promotes mitochondrial function and oxidative fiber formation, whereas PGC-1α4 facilitates muscle hypertrophy by inducing insulin-like growth factor 1 (IGF-1) and repressing myostatin [11,12]. In white adipose tissue (WAT), PGC-1α facilitates the browning process through PR domain containing 16 (PRDM16) and uncoupling protein 1 (UCP-1), leading to the formation of beige adipocytes with elevated mitochondrial activity [13,14]. Furthermore, thermogenic gene expression in brown adipose tissue (BAT), including mitochondrial transcription factor A (Tfam) and UCP-1, is also regulated by PGC-1α [14]. Given its dual regulatory role in both skeletal muscle and adipose tissue, targeting PGC-1α represents a promising therapeutic strategy for combating sarcopenic obesity. Accordingly, increasing attention has been directed toward natural compounds capable of modulating PGC-1α signaling.

**Figure 1:**
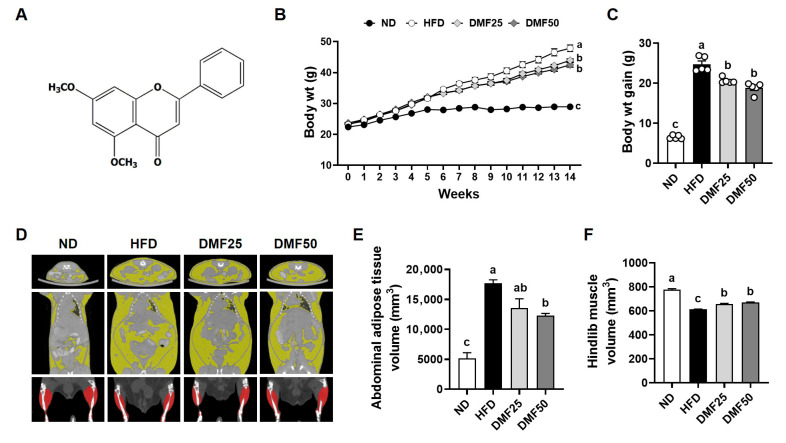
Effects of DMF on body weight and the volumes of adipose tissue and skeletal muscle in HFD-induced obese mice. (**A**) Chemical structure of DMF. (**B**) Body weight was recorded weekly over a 14-week period. (**C**) Total body weight gain at the end of the experimental period. (**D**,**E**) Abdominal adipose tissue volume and (**F**) hindlimb muscle volume were assessed using micro-CT; *n* = 4–5 per group. Data are expressed as the mean ± SEM. Distinct letters above the bars indicate significant differences between groups at *p* < 0.05.

5,7-Dimethoxyflavone (DMF, Figure 1A), a bioactive flavonoid found in *Kaempferia parviflora* (black ginger) has demonstrated multiple health benefits, including anti-obesity, anti-inflammatory, and muscle-protective effects [15,16,17]. DMF has been shown to regulate lipid metabolism in the adipose tissue of high-fat-diet (HFD)-induced obese mice and enhance mitochondrial biogenesis in the skeletal muscle of aged sarcopenic mice through the activation of PGC-1α-related pathways [1,18,19]. However, the therapeutic potential of DMF in sarcopenic obesity has not been fully investigated. Importantly, sarcopenic obesity involves a pathological interaction between adipose and muscle tissues, where excess fat accelerates muscle loss via inflammation and metabolic disruption, underscoring the need for an integrated evaluation of both tissues [9]. While previous studies have focused on either adipose or muscle tissue in isolation, the present study uniquely investigates the dual regulatory effects of DMF on both skeletal muscle and adipose tissue under sarcopenic obese conditions. We hypothesized that DMF mitigates sarcopenic obesity by reducing fat mass and enhancing skeletal muscle mass through improved mitochondrial function via PGC-1α-mediated pathways. To test this hypothesis, we employed an HFD-induced obese mouse model and comprehensively examined molecular and phenotypic alterations in the skeletal muscle and WAT.

## 2. Materials and Methods

### 2.1. Chemical Reagents

DMF was obtained from Aktin Chemicals, Inc. (Chengdu, China). 2,2,2-Tribromoethanol and the protease inhibitor cocktail were procured from Sigma-Aldrich (St. Louis, MO, USA). RT-Premix, 5× sample buffer, and NP-40 lysis buffer were purchased from Elpis Biotech (Daejeon, Republic of Korea). 5× Loading star and Tris-buffered saline (pH 7.5) were supplied by DyneBio (Gyeonggi, Republic of Korea). Primary antibodies against PI3K, phospho (p)-PI3K, Akt, p-Akt, forkhead box O3 (FoxO3), p-FoxO3, mammalian target of rapamycin (mTOR), p-mTOR, eukaryotic initiation factor 4E binding protein 1 (4EBP1), p-4EBP1, 70-kDa ribosomal protein S6 kinase (P70S6K), and p-P70S6K, as well as α-tubulin, were purchased from Cell Signaling Technology (Beverly, MA, USA). Horseradish-peroxidase-conjugated goat anti-rabbit antibody and SafeDry Taq PCR premix were purchased from Bethyl Laboratories, Inc. (Montgomery, TX, USA) and CellSafe (Gyeonggi, Republic of Korea), respectively.

### 2.2. Animal Experiments

Four-week-old male C57BL/6J mice (Daehan Biolink Co., Eumsung, Republic of Korea) were housed in a climate-controlled room (12-12 h dark–light cycle and 25 °C) and had access to water ad libitum. After 1 week of acclimation, 20 total mice were divided into weight-matched groups: (1) normal diet (ND, control, *n* = 5), (2) HFD (HFD-induced sarcopenic obesity group, *n* = 5), (3) DMF25 (HFD plus 25 mg/kg/day-treated group, *n* = 5), (4) DMF50 (HFD plus 50 mg/kg/day-treated group, *n* = 5). The sample size was calculated using G*Power 3.1.9.4 for one-way ANOVA (f = 1.092, α = 0.05, power = 0.80) based on previous studies [19,20], yielding 16 mice (4 per group). One extra mouse per group was added to account for potential failure, resulting in 20 mice (5 per group). Mice in the ND group were fed a diet containing 10% kcal fat content (Rodent diet D12450B; Research Diets, New Brunswick, NJ, USA), while those in the HFD, DMF25, and DMF50 groups were fed a diet with a 60% kcal fat content (Rodent diet D12492; Research Diets) for 14 weeks. After 6 weeks of the HFD diet, DMF was administered daily via oral gavage at doses of 25 mg/kg/day or 50 mg/kg/day to the DMF25 and DMF50 groups, respectively, for the remaining 8 weeks. To minimize confounders, cages were alternately arranged among groups, and all treatments and measurements were conducted in a consistent order. Body weight and food intake were measured weekly. At the end of the experimental period, animals were anesthetized intraperitoneally with 350 mg/kg 2,2,2-tribromoethanol, and blood samples were collected via cardiac puncture. Subsequently, skeletal muscles, including the gastrocnemius (GA), tibialis anterior (TA), extensor digitorum longus (EDL), and soleus (SOL), along with WAT, including subcutaneous (sWAT), perirenal (pWAT), and epididymal (eWAT) fat, and interscapular brown adipose tissue (BAT), were carefully dissected and weighed. GA muscle, sWAT, and BAT were divided in half, with one portion fixed in 10% formalin and the remainder stored at −80 °C for further analysis. All the experimental procedures were reviewed and approved by the Institutional Animal Care and Use Committee (IACUC) of Yonsei University (Seoul, Republic of Korea) (Permit number: IACUC-A-201905-905-02). Blinding was not implemented due to the need for daily oral administration, body weight monitoring, and behavioral testing, which required direct investigator involvement. Humane endpoints were not established, as the experimental protocol was not expected to cause substantial pain or distress. Animals were routinely monitored during daily oral administration to assess the general health status. All experimental procedures were performed in accordance with institutional animal care guidelines to minimize potential discomfort and stress.

### 2.3. Treadmill Test

An exercise endurance test was conducted using a treadmill apparatus (LE8710MTS; Panlab, Barcelona, Spain). The protocol began with a speed of 10 cm/s for 10 min, after which the speed was increased by 1 cm/s every minute until reaching 30 cm/s. This maximum speed was then maintained for the remainder of the test. The time and distance at which each mouse reached exhaustion were recorded. Exhaustion was defined as the inability to continue running for 10 s despite receiving a 0.2 mA electric stimulus, which was applied solely as negative reinforcement and did not cause harm to the animals.

### 2.4. Grip Strength Test

Forelimb and fore/hindlimb grip strength tests were performed using a grip strength meter (Panlab). Each mouse was gently placed on a grid, and after confirming that it had firmly grasped the grid, its tail was carefully pulled backward. Once the mouse released its grip, the recorded value was noted as the grip strength. Each mouse underwent six consecutive trials, and the mean value was calculated to represent the grip strength for that individual.

### 2.5. Micro-Computed Tomography (CT) Imaging

Micro-CT experiments were conducted to measure muscle and fat volumes using a positron emission tomography (PET)/CT/single-photon emission tomography (SPECT) system (Siemens Inveon, Knoxville, TN, USA). CT images were further analyzed using the Inveon Research Workplace software (version 4.2, Siemens Inveon).

### 2.6. Histological Analysis

Paraffin sections were prepared from fixed GA muscle, sWAT, and BAT and subsequently stained with hematoxylin and eosin (H&E). Stained tissue areas were visualized and randomly imaged using a CK40 inverted microscope (Olympus, Tokyo, Japan) equipped with a T500 camera (eXcope, Daejeon, Republic of Korea). The mean cross-sectional area (CSA) of GA muscle fibers and the average sizes of sWAT and BAT adipocytes were quantified using ImageJ software (version 1.47, National Institutes of Health, Bethesda, MD, USA).

### 2.7. Western Blot Analysis

Proteins were extracted from GA muscle tissues with NP-40 lysis buffer containing a protease inhibitor cocktail. Equal amounts of protein from each sample were denatured in 5× sample buffer at 95 °C for 5 min. The samples were then separated via 10% sodium dodecyl sulphate-polyacrylamide gel electrophoresis (SDS-PAGE) at 85 V for 30 min followed by 110 V for 90 min. Subsequently, proteins were transferred onto 0.45 μm nitrocellulose membranes (GE Healthcare, Piscataway, NJ, USA), which were then blocked with 2.5% skimmed milk in Tris-buffered saline containing Tween 20 (TBST) for 30 min. The membranes were incubated with primary antibodies at 4 °C for 18 h, followed by secondary antibodies at 4 °C for 2 h. Primary and secondary antibodies were diluted 1:1000 and 1:5000, respectively, in TBST. Protein bands were detected using an enhanced chemiluminescence solution (Amersham Biosciences, Little Chalfont, UK) and visualized with the G:BOX EF imaging system (Syngene, Cambridge, UK) using the Gene Snap program (version 1.3.9.0). Band intensities were quantified densitometrically using ImageJ software (National Institutes of Health).

### 2.8. Reverse Transcription-Polymerase Chain Reaction (RT-PCR)

Total RNA was extracted from GA muscle tissues, sWAT, and BAT using TRIzol reagent (Takara, Otsu, Japan). The RNA concentration was measured with a NanoDrop Lite spectrophotometer (Thermo Fisher Scientific Inc., Waltham, MA, USA), and samples with 260/280 ratios greater than 1.8 were used for cDNA synthesis. cDNA was generated using RT-Premix on a SimpliAmp thermal cycler (Applied Biosystems, Foster City, CA, USA) at 42 °C for 60 min, followed by 95 °C for 5 min. PCR amplification was performed with SafeDry Taq PCR premix and target gene primers (Bioneer, Daejeon, Republic of Korea). The thermal cycling conditions included an initial activation at 95 °C for 5 min, followed by 35 cycles of 95 °C for 30 s, 58 °C for 30 s, and 72 °C for 45 s. PCR products were stained with 5× Loading star and separated via 1.5% agarose gel electrophoresis. The bands were visualized using a G:BOX EF imaging system (Syngene) and the Gene Snap software (Syngene), and densitometric analysis was performed using ImageJ software (National Institutes of Health). Mitochondrial content was assessed by calculating the mitochondrial DNA (mtDNA)-to-genomic DNA (gDNA) ratio based on the relative band intensities.

### 2.9. Adenosine Triphosphate (ATP) Measurement

SOL muscle tissues were homogenized in ice-cold 2 N perchloric acid and incubated on ice for 30 min. After centrifugation (13,000× *g*, 4 °C, 2 min), supernatants were neutralized with 2 N KOH (pH 6.5–8.0) and centrifuged again (13,000× *g*, 4 °C, 15 min) to remove precipitates. ATP levels were determined using a commercial ATP assay kit (Biomax, Gyeonggi-do, Republic of Korea), and absorbance was measured at 570 nm with a microplate reader (VersaMax™, Molecular Devices, San Jose, CA, USA).

### 2.10. Statistical Analysis

Group comparisons were analyzed by applying one-way analysis of variance (ANOVA), with Tukey’s multiple comparison test used for post hoc analysis as appropriate. For certain comparisons, unpaired *t*-tests were conducted. All statistical analyses were carried out using GraphPad Prism version 10.0 (GraphPad Software, La Jolla, CA, USA). Outliers were identified using the GraphPad outlier calculator before analysis. Results were considered statistically significant at *p* < 0.05, and values are presented as the mean ± standard error of the mean (SEM).

## 3. Results

### 3.1. DMF Reduced Weight Gain and Visceral Abdominal Fat, While Enhancing Hindlimb Muscle Volume in Obese Mice

Following 8 weeks of oral administration, DMF25 and DMF50 groups showed significantly lower final body weights and weight gain than the HFD group (Figure 1B,C), with no changes in food intake. Given the importance of both fat and muscle accumulation in sarcopenic obesity, micro-CT analysis was performed to assess the distribution of white adipose and muscle tissues in mice (Figure 1D). The volume of visceral abdominal fat was significantly higher in the HFD group compared to the ND group (Figure 1E). However, DMF administration markedly reduced fat accumulation in the visceral abdominal region, as observed in both the DMF25 and DMF50 groups. The total abdominal adipose tissue volumes were 5142.23 ± 1659.61 mm^3^ (ND), 17,687.30 ± 996.58 mm^3^ (HFD), 13,571.85 ± 3063.86 mm^3^ (DMF25), and 12,270.87 ± 655.19 mm^3^ (DMF50), respectively. Conversely, hindlimb muscle volume in the HFD group was significantly diminished by 20.9% compared to the ND group (Figure 1F). However, DMF administration significantly restored the muscle volume, with increases of approximately 9% observed in both the DMF25 and DMF50 groups. The hindlimb muscle volumes were 776.53 ± 14.91 mm^3^ (ND), 613.90 ± 2.86 mm^3^ (HFD), 658.33 ± 7.72 mm^3^ (DMF25), and 669.30 ± 7.95 mm^3^ (DMF50), respectively.

### 3.2. DMF Enhanced Muscle Mass and Myofiber CSA, Improving Grip Strength and Exercise Capacity in Obese Mice

**Figure 2:**
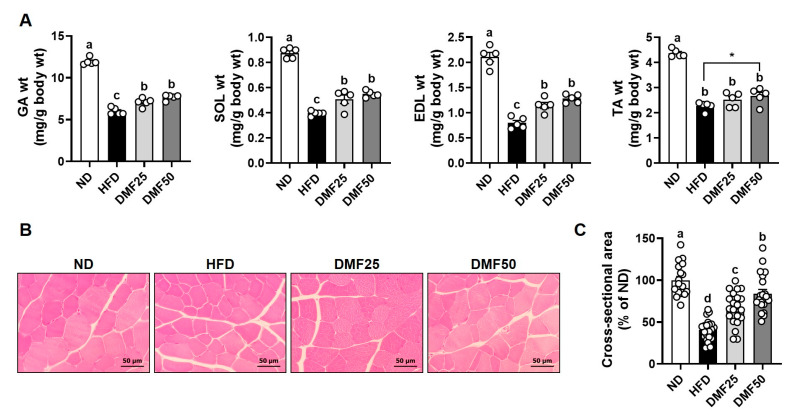
Effects of DMF on muscle weights and myofiber CSA in HFD-induced obese mice. (**A**) Weights of the GA, SOL, EDL, and TA muscles. (**B**,**C**) Representative H&E-stained images of GA muscle and quantification of myofiber CSA; *n* = 5 per group. Data are expressed as the mean ± SEM. Statistically significant differences between the two groups are indicated by asterisks (*), based on unpaired *t*-test analysis (*p*  <  0.05). Distinct letters above the bars indicate significant differences between groups at *p* < 0.05.

**Figure 3:**
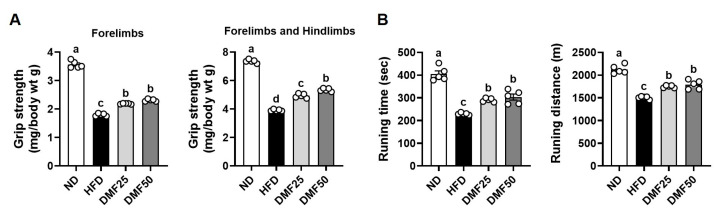
Effects of DMF on grip strength and exercise capacity in HFD-induced obese mice. (**A**) Forelimb and combined fore/hindlimb grip strengths assessed using a grip strength meter. (**B**) Running time and distance measured through treadmill testing; *n* = 5 per group. Data are expressed as the mean ± SEM. Distinct letters above the bars indicate significant differences between groups at *p* < 0.05.

Sarcopenic obesity manifests as a decline in skeletal muscle mass and function, primarily attributed to the deleterious effects of excessive adipose tissue accretion [6]. To assess this condition, we measured individual muscle weights, myofiber CSA, grip strength, and exercise capacity as indicators of muscle quality and function. The weights of the GA, SOL, EDL, and TA muscles were significantly reduced in the HFD group compared to the ND group. However, DMF administration significantly attenuated this muscle weight loss, with the DMF50 group exhibiting respective increases of 28.0%, 38.7%, 61.2%, and 17.9% in GA, SOL, EDL, and TA muscles, respectively (Figure 2A). Consistently, myofiber CSA was significantly decreased by 59.4% in the HFD group compared to the ND group. However, DMF administration significantly improved the CSA, with increases of 25.7% and 43.4% observed in the DMF25 and DMF50 groups, respectively (Figure 2B). Given the established association between muscle atrophy and functional decline, muscle strength and endurance were assessed prior to sacrifice. Specifically, grip strength served as an indicator of resistance capacity, while treadmill running quantified the aerobic capacity. HFD notably decreased the forelimb grip strength by 12.05% and combined fore/hindlimb grip strength by 16.47% compared to the ND group (Figure 3A). In contrast, DMF administration significantly enhanced grip strength in both the DMF25 and DMF50 groups. The running distance and time were reduced in the HFD group compared to the ND group. However, DMF administration significantly improved exercise performance, as evidenced by increases in the running distance, by 26.54% and 32.03%, and in the running time, by 15.99% and 18.88%, in the DMF25 and DMF50 groups, respectively (Figure 3B).

### 3.3. DMF Regulated the Expression of Key Markers Involved in Protein Degradation and Synthesis in the GA Muscle of Obese Mice

**Figure 4:**
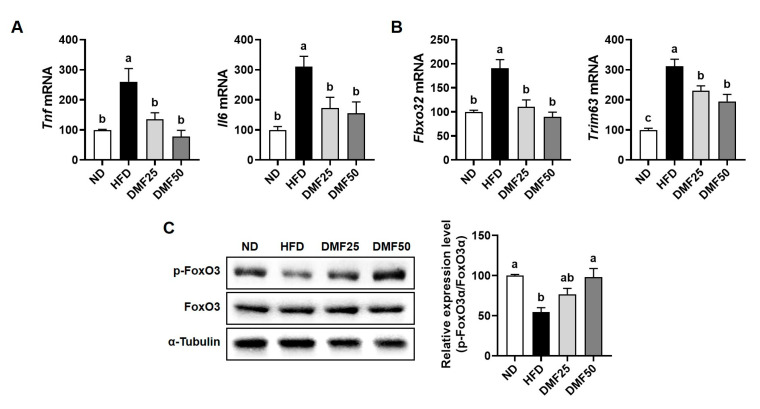
Effects of DMF on inflammatory cytokines and protein degradation-related markers in the GA muscle of HFD-induced obese mice. (**A**) The mRNA expression levels of inflammatory cytokines, (**B**) *Fbxo32* (MuRF1), and *Trim63* (atrogin-1) analyzed via RT-PCR. (**C**) Protein expression levels of FoxO3 phosphorylation assessed via Western blotting; *n* = 3–5 per group. Data are expressed as the mean ± SEM. Distinct letters above the bars indicate significant differences between groups at *p* < 0.05.

**Figure 5:**
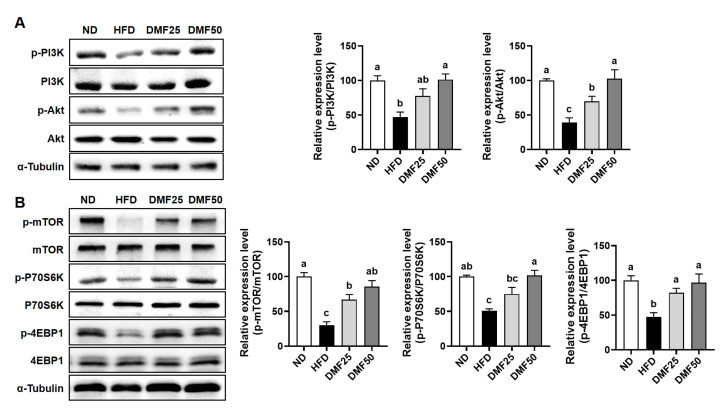
Effects of DMF on protein synthesis-related markers in the GA muscle of HFD-induced obese mice. (**A**) Protein expression levels of phosphorylated PI3K and Akt assessed via Western blotting. (**B**) Protein levels of phosphorylated mTOR, P70S6K, and 4EBP1 assessed via Western blotting; *n* = 3 per group. Data are expressed as the mean ± SEM. Distinct letters above the bars indicate significant differences between groups at *p* < 0.05.

Sarcopenic obesity is primarily characterized by a significant decrease in skeletal muscle mass, largely due to the complex interaction of reduced protein synthesis and increased protein degradation [21]. Therefore, we evaluated whether DMF could attenuate the increased protein degradation and restore protein synthesis-related marker expression in the GA muscle of obese mice. HFD significantly increased the gene expression of inflammatory cytokines, such as *Tnf* and *Il6*, compared to the ND group, while DMF administration notably suppressed this increase, restoring expression levels to those similar to the ND group (Figure 4A). Also, the gene expression of muscle-specific E3 ubiquitin ligases, including atrogin-1 (gene name *Fbxo32*) and MuRF1 (gene name *Trim63*), was higher in the HFD group than in ND, but significantly reduced by DMF in both DMF25 and DMF50 groups (Figure 4B). Furthermore, the protein expression of phosphorylated FoxO3α, a transcription factor that regulates muscle-specific E3 ubiquitin ligases, was reduced in the HFD group compared to the ND group (Figure 4C). However, DMF administration markedly restored its expression in a dose-dependent manner in both the DMF25 and DMF50 groups, with levels comparable to those of the ND group. The PI3K/Akt and mTOR/P70S6K/4EBP1 signaling pathways play a crucial role in regulating muscle protein synthesis, a process that becomes disrupted in obese sarcopenia [21]. The protein expression levels of phosphorylated PI3K and Akt were significantly decreased in the HFD group compared to the ND group. However, DMF administration dose-dependently increased the phosphorylation of both proteins, with the DMF50 group showing levels comparable to the ND group (Figure 5A). Similarly, the phosphorylation levels of mTOR and its downstream targets, p70S6K and 4EBP1 were markedly reduced in the HFD group (Figure 5B). DMF administration notably restored the phosphorylation of protein synthesis markers, especially in the DMF50 group, which showed phosphorylated levels of PI3K/Akt and mTOR/P70S6K/4EBP1 that were almost similar to those in the ND group.

### 3.4. DMF Increased the Gene Expression of Mitochondrial Function- and Muscle Growth-Related Markers in the GA Muscle of Obese Mice

**Figure 6:**
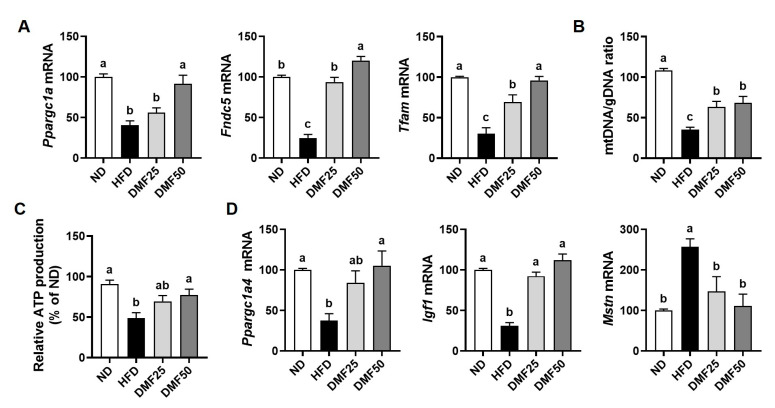
Effects of DMF on the expression of PGC-1α isoforms and related markers in the GA muscle of HFD-induced obese mice. (**A**) The mRNA expression levels of *Ppargc1a1*, *Fndc5*, and *Tfam* analyzed via RT-PCR. (**B**) Relative mitochondrial DNA content determined based on the mtDNA/gDNA ratio. (**C**) ATP production in GA muscle tissue. (**D**) The mRNA expression levels of *Ppargc1a4*, *Igf1*, and *Mstn* analyzed via RT-PCR; *n* = 5 per group. Data are expressed as the mean ± SEM. Distinct letters above the bars indicate significant differences between groups at *p* < 0.05.

Sarcopenic PGC-1α1 and PGC-1α4 are known to be essential regulators of mitochondrial function and muscle hypertrophy, respectively, and their impaired activity contributes to the progression of sarcopenic obesity [11]. To determine the impact of DMF on these pathways, we evaluated the gene expression of PGC-1α1, PGC-1α4, and their downstream targets in the GA muscle. Compared to the ND group, the expression of *Ppargc1a* (gene name PGC-1α1), fibronectin type III domain-containing protein (*Fndc5*), and *Tfam* was significantly lower in the HFD group. However, DMF administration restored these gene expression levels in a dose-dependent manner, reaching levels similar to those in the ND group (Figure 6A). Since Tfam is involved in mitochondrial DNA replication and transcription, we measured the mtDNA/gDNA ratio to evaluate mitochondrial biogenesis and functionality. HFD led to a reduction in the mtDNA/gDNA ratio, which was significantly elevated after DMF administration (Figure 6B). We further assessed mitochondrial functional output, and intracellular ATP levels were measured. ATP production was notably lower in the HFD group compared to the ND group, while DMF treatment significantly raised ATP levels (Figure 6C). The DMF50 group exhibited ATP levels comparable to those of the ND group, indicating the functional recovery of mitochondrial energy production. Furthermore, the gene expression levels of *Ppargc1a4* and *Igf1*, both associated with muscle hypertrophy, were significantly diminished in the HFD group but were significantly restored after DMF administration (Figure 6D). Conversely, *Mstn* (gene name myostatin), a negative regulator of muscle mass, was elevated in HFD mice but reduced after DMF administration to levels similar to ND.

### 3.5. DMF Alleviated Adiposity and Promoted Gene Expression Linked to Beige Adipocyte Formation in sWAT of Obese Mice

**Figure 7:**
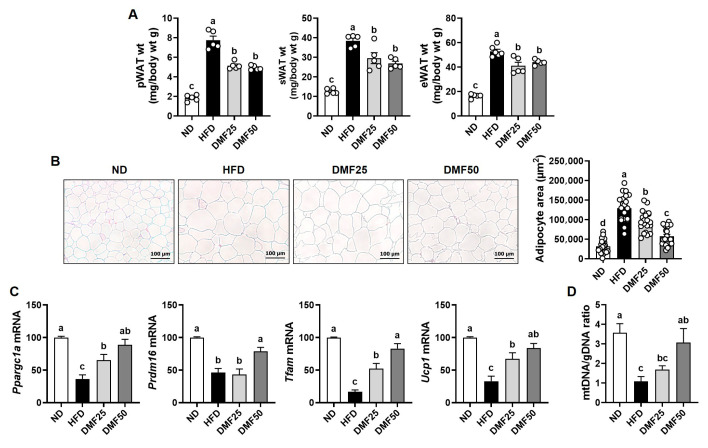
Effects of DMF on beige adipocyte formation in sWAT of HFD-induced obese mice. (**A**) Weights of pWAT, sWAT, and eWAT. (**B**) Adipocyte size in sWAT assessed via H&E staining (magnification, 100×). (**C**) The mRNA expression levels of *Ppargc1*, *Prdm16*, and *Ucp1* analyzed via RT-PCR. (**D**) Relative mitochondrial DNA content determined based on the mtDNA/gDNA ratio; *n* = 5 per group. Data are expressed as the mean ± SEM. Distinct letters above the bars indicate significant differences between groups at *p* < 0.05.

The weights of pWAT, sWAT, and eWAT were significantly higher in the HFD group compared to the ND group, whereas DMF administration significantly reduced all types of WAT weights (Figure 7A). As shown via H&E staining analysis, adipocyte size in sWAT was markedly increased in the HFD group, whereas DMF administration dose-dependently attenuated adipocyte hypertrophy (Figure 7B). Beige adipocytes are known to alleviate sarcopenic obesity by promoting energy expenditure and reducing adipose tissue inflammation [22]. Therefore, to evaluate beige adipocyte induction, we analyzed the expression of beige adipocyte-related genes in sWAT. *Ppargc1a*, *Prdm16*, *Ucp1*, and *Tfam* are key regulators of mitochondrial biogenesis and thermogenic activation during beige adipocyte formation. The expression of these genes was significantly lower in the HFD group compared to the ND group, while DMF administration dose-dependently restored their expression to levels similar to those of the ND group (Figure 7C). Furthermore, the reduction in the mtDNA/gDNA ratio induced by the HFD was significantly restored after DMF administration, but this effect was observed only in the DMF50 group, not in the DMF25 group (Figure 7D).

### 3.6. DMF Enhanced Thermogenic Gene Expression and Mitochondrial Activity in BAT of Obese Mice

**Figure 8:**
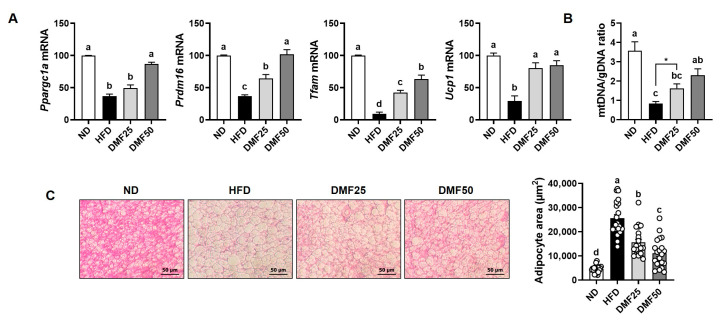
Effects of DMF on thermogenic activation in BAT of HFD-induced obese mice. (**A**) The mRNA expression levels of *Ppargc1a*, *Prdm16*, *Tfam*, and *Ucp1* analyzed via RT-PCR. (**B**) Relative mtDNA content determined based on the mtDNA/gDNA ratio. (**C**) Adipocyte size in BAT evaluated via H&E staining (magnification, 200×); n = 5 per group. Data are expressed as the mean ± SEM. Statistically significant differences between the two groups are indicated by asterisks (*), based on unpaired *t*-test analysis (*p*  <  0.05). Distinct letters above the bars indicate significant differences between groups at *p* < 0.05.

As BAT is characterized by its thermogenic capacity mediated by UCP-1 [23,24], we examined the expression of thermogenesis-related genes in BAT to assess the effect of DMF on BAT activation. The gene expression levels of *Ppargc1a*, *Prdm16*, *Ucp1*, and *Tfam* were significantly lower in the HFD group compared to the ND group, while DMF administration restored their expression in a dose-dependent manner (Figure 8A). Consistently, the relative mtDNA content in the HFD group was lower than in the ND group (Figure 8B). However, DMF administration significantly increased the mtDNA/gDNA ratio in BAT. Histological analysis revealed that adipocyte size in the BAT was enlarged in the HFD group, but DMF treatment significantly decreased adipocyte hypertrophy (Figure 8C).

## 4. Discussion

Sarcopenic obesity is a pathological condition characterized by the coexistence of excessive fat accumulation and progressive skeletal muscle loss, accompanied by a decline in physical function [3]. Globally, it has been reported that approximately 11% of individuals aged 60 years and older are affected by sarcopenic obesity, with the prevalence increasing to 23% among those aged 75 and above [25,26]. In the present study, we found that HFD-fed mice exhibited hallmark features of sarcopenic obesity, including increased body weight and adipose tissue mass, as well as reduced skeletal muscle mass, muscle volume, grip strength, and exercise capacity. However, the administration of DMF markedly improved the sarcopenic obesity-related pathology by restoring muscle mass and function, increasing muscle volume, and reducing both body weight and epididymal and perirenal WAT mass. These results suggest that DMF effectively attenuates HFD-induced sarcopenic obesity by improving both muscle and adipose tissue homeostasis. Our previous studies support the current findings by demonstrating that DMF suppresses adipogenesis and fat accumulation in HFD-induced obese mice [1] and enhances muscle mass and function in aged mice with sarcopenia [19]. However, despite these results, the effect of DMF on sarcopenic obesity, a condition characterized by the coexistence of obesity and muscle wasting, has not been fully explored. The present study provided the first experimental evidence that DMF can simultaneously mitigate both obesity and muscle atrophy in a sarcopenic obesity model. Taken together, these data indicate that DMF exerts both anti-obesity and muscle-preserving effects and may therefore serve as a potential therapeutic candidate for the management of sarcopenic obesity. Nevertheless, further clinical investigations will be necessary to validate these findings in human populations and to clarify the underlying mechanisms of DMF in the context of sarcopenic obesity.

Sex hormones such as estrogen and testosterone are known to differentially affect muscle mass, fat distribution, and inflammatory responses [27], suggesting that the physiological effects of DMF may vary between sexes. The present study was conducted exclusively using male mice to minimize the variability associated with the female estrous cycle, which can influence metabolic homeostasis, muscle regeneration, and mitochondrial function. Previous studies have reported sex-specific outcomes in metabolic and muscular disorders [27]. Moreover, *Kaempferia parviflora* extract, a natural source of DMF, has been shown to increase testosterone levels in mice, indicating possible sex-related interactions [28]. Therefore, further research using female models, including both intact and ovariectomized mice, is important to determine whether DMF has sex-dependent effects on mitochondrial function and muscle metabolism.

Previous studies indicate that administering DMF orally at 10 to 30 mg/kg and intravenously at up to 50 mg/kg was well tolerated in rodents, with no signs of toxicity, highlighting its good safety profile [29,30]. Also, pharmacokinetic data from the same study showed that after the oral administration of 10 mg/kg DMF, the compound reached a *C*_max_ of 1180 ng/g and an AUC_t_ of 345 h·ng/g in skeletal muscle, indicating moderate tissue exposure [29]. Based on this evidence, it is likely that the higher doses used in this study, DMF 25 and DMF 50 mg/kg, led to enough skeletal muscle accumulation to produce the observed protective effects against muscle atrophy. Furthermore, DMF has a relatively low oral bioavailability of 2% when administered to rats at a dose of 250 mg/kg of *Kaempferia parviflora* ethanol extract containing 21.1 mg/g DMF [30]. Nevertheless, its extensive tissue distribution, including the liver, kidney, brain, and adipose tissues, suggests the potential for off-target effects, which should be considered in future studies. Notably, clinical studies have shown that consuming a standardized *Kaempferia parviflora* extract (containing 2–5% DMF) at doses of 90–100 mg in humans increased overall energy expenditure, enhanced physical fitness and oxidative status, and improved self-reported sexual health without evidence of toxicity [16]. These findings support the potential translational relevance of DMF for human use. However, further clinical studies are needed to confirm the safety and effectiveness of DMF in humans.

PGC-1α is known to be a master regulator of mitochondrial biogenesis and metabolic activity in both skeletal muscle and adipose tissue [11]. In skeletal muscle, the upregulation of PGC-1α1 increases the number and function of mitochondria. This occurs through the stimulation of Tfam-mediated mitochondrial DNA replication, oxidative phosphorylation, angiogenesis, and fatty acid oxidation. Together, these processes contribute to improved endurance capacity and offer protection against the sarcopenia and metabolic dysfunction that can occur with aging [31]. In this study, we found that DMF significantly elevated the expression of *Ppargc1a* and *Tfam*, accompanied by increased mtDNA content in the GA muscle of HFD mice, indicating enhanced mitochondrial biogenesis. This upregulation likely promoted ATP production via improved oxidative capacity, thereby contributing to the observed improvements in exercise capacity and grip strength. On the other hand, in WAT, PGC-1α also plays a critical role in regulating energy metabolism by inducing mitochondrial biogenesis through Tfam, promoting UCP-1-mediated thermogenesis, and facilitating the PRDM16-dependent browning of white adipocytes into beige adipocytes [14]. These processes increase energy expenditure, improve glucose homeostasis, reduce inflammation, and attenuate obesity-related metabolic dysfunction. In this study, we observed that DMF increased the mRNA expression levels of *Ppargc1a*, *Prdm16*, *Ucp1*, and *Tfam*, while also elevating beige adipocyte markers and mtDNA content in sWAT. DMF reduced adipocyte size in sWAT and decreased fat mass in both the epididymal and perirenal regions. Furthermore, the mitochondrial activation induced by DMF led to increased ATP production in adipocytes, which likely contributes to enhanced thermogenesis and metabolic activity in WAT. By upregulating PGC-1α in both skeletal muscle and adipose tissue, DMF appears to rejuvenate mitochondrial function and enhance systemic energy metabolism. This activation promotes oxidative capacity in muscle and facilitates adaptive browning and thermogenic activation in WAT. Consequently, DMF administration alleviated muscle wasting and excessive fat accumulation in HFD-fed mice, primarily by enhancing PGC-1α activation in conjunction with increased mitochondrial function, thereby ameliorating the key features of sarcopenic obesity. However, since it was not directly established that causality exists between DMF and PGC-1α activation, further investigations employing the knockdown or inhibition of PGC-1α or FNDC5 are necessary to clarify the underlying mechanisms.

PGC-1α4, a splice variant of PGC-1α, promotes muscle hypertrophy by increasing IGF-1 expression and suppressing myostatin, which in turn activates the PI3K/Akt signaling pathway, a key regulator of muscle protein synthesis and growth [11,12]. In the present study, we found that DMF administration significantly stimulated the gene expression of *Ppargc1a4* and *Igf1*, while suppressing *Mstn* gene expression in HFD-induced obese mice. Furthermore, this was accompanied by increased phosphorylation of PI3K and Akt, suggesting that DMF activates anabolic signaling through the PGC-1α4, IGF-1, and Akt axis. Consistently, DMF also activated the mTOR signaling pathway downstream of Akt, as indicated by the increased phosphorylation of mTOR, P70S6K, and 4EBP1. In parallel, DMF inhibited FoxO3 nuclear translocation by promoting its phosphorylation, thereby reducing the expression of the muscle-specific E3 ligases. These results indicate that DMF simultaneously enhances protein synthesis and suppresses protein degradation, leading to improved muscle mass. While our previous study demonstrated that DMF activates mTOR signaling in aged mice, the current study uniquely identifies PGC-1α4 as an upstream regulator in the context of sarcopenic obesity. These findings are further supported by evidence that α-cedrene, a plant-derived sesquiterpene, increases muscle mass in healthy mice through activation of the PGC-1α4/PI3K/Akt signaling pathway [12], providing mechanistic support for the role of PGC-1α4 in muscle hypertrophy. Extending these insights, our study provides novel evidence that DMF preserves muscle mass in metabolically compromised conditions by upregulating PGC-1α4 and coordinately regulating both anabolic and anti-catabolic signaling cascades.

The activation of BAT is known to depend on key regulators such as PRDM16, UCP-1, Tfam, and PGC-1α. Although we did not observe any significant changes in the weight of BAT, DMF significantly increased the content of mtDNA and the mRNA expression levels of *Ppargc1a*, *Prdm16*, *Ucp1*, and *Tfam* in the BAT of obese mice fed an HFD. In brown adipocytes, PGC-1α functions as a central upstream regulator orchestrating both mitochondrial biogenesis and thermogenic gene expression [32]. Specifically, PGC-1α enhances thermogenesis by inducing UCP-1, which uncouples mitochondrial respiration to generate heat, and upregulates PRDM16, a transcriptional coactivator critical for brown adipocyte identity. It also promotes mitochondrial biogenesis by activating Tfam, a nuclear-encoded factor essential for mtDNA replication and transcription [32]. Supporting this dual role, studies in HFD-induced obese mice have shown that PGC-1α overexpression stimulates brown fat thermogenesis and mitochondrial oxidative metabolism, leading to increased energy expenditure and protection against diet-induced obesity [33]. Our findings are consistent with this, as DMF administration in HFD-fed mice increased mtDNA content and the expression of *Ppargc1a*, *Prdm16*, *Ucp1*, and *Tfam* in BAT, indicating enhanced mitochondrial content and thermogenic capacity.

We found that DMF has diverse regulatory effects on skeletal muscle, BAT, and WAT in a model of HFD-induced sarcopenic obesity. However, the potential mechanisms through which DMF coordinates metabolic regulation across these tissues within a single organism remain largely unclear. Future studies are warranted to investigate the molecular crosstalk between skeletal muscle and adipose tissues in response to DMF. One possible explanation is that DMF may exert its systemic effects through the myokine irisin, a cleaved and secreted form of FNDC5. PGC-1α1 not only promotes mitochondrial biogenesis in skeletal muscle by upregulating Tfam but also induces the transcription of FNDC5 [10]. Circulating irisin, released from skeletal muscle, has been shown to affect multiple target tissues, including the brain, liver, and adipose tissue [34]. Notably, irisin plays a key role in promoting the browning of WAT by stimulating the formation of beige adipocytes [13,34]. In this study, DMF increased the mRNA expression levels of both *Ppargc1a* and *Fndc5* in the GA muscle of HFD-induced obese mice, suggesting that DMF may enhance irisin secretion, thereby promoting thermogenesis and lipid metabolism in adipose tissues. Another possible mechanism is that DMF may act, at least in part, through β-adrenergic signaling. In addition to promoting muscle hypertrophy [12], β-adrenergic receptor activation is known to stimulate lipolysis and thermogenesis in BAT [32]. These effects are primarily mediated by the cyclic adenosine monophosphate (cAMP) signaling pathway, which functions as a secondary messenger in both skeletal muscle and BAT. Previous studies have shown that DMF dose-dependently increases intracellular cAMP levels in B16F10 cells [35], suggesting that DMF may influence β-adrenergic receptor-mediated signaling pathways. Together, these mechanisms may underlie the integrated effects of DMF across muscle and adipose tissues.

## 5. Conclusions

In conclusion, we demonstrated that DMF attenuates the progression of sarcopenic obesity in HFD-fed sarcopenic obese mice. DMF administration led to significant reductions in body weight and adipose tissue weight and volume, while simultaneously improving grip strength, running capacity, and muscle mass and volume. At the molecular level, DMF increased the gene expression of both *Ppargc1a* and *Ppargc1a4*, thereby promoting mitochondrial biogenesis and regulating protein turnover through the coordinated modulation of protein synthesis and degradation pathways. Additionally, DMF increased the gene expression of *Ppargc1a*, *Prdm16*, *Ucp1*, and *Tfam* and elevated the relative mtDNA content and ATP production in both WAT and BAT, indicating the induction of beige adipocyte formation and the thermogenic activation of BAT. Taken together, these findings suggest that DMF has promising therapeutic potential for the management of sarcopenic obesity by targeting both muscle loss and adiposity. However, further studies are needed to assess its safety, efficacy, and translational applicability in humans.

## Data Availability

The original contributions presented in the study are included in the article; further inquiries can be directed at the corresponding authors.
